# Coordination and collaboration for strengthening respiratory surveillance at the national level: updates from workshop hosted by the WHO Hub for Pandemic and Epidemic Intelligence, 24–25 July 2024

**DOI:** 10.1186/s12919-025-00341-5

**Published:** 2025-09-08

**Authors:** Raquel Medialdea Carrera, Aspen Hammond, Jijoho Mischael Michel, Hannah Lewis, Yeon Kyeng Lee, Lidia Alexandrova Ezerska, George Sie Williams, Wenqing Zhang, Philip AbdelMalik

**Affiliations:** 1 World Health Organization Hub for Pandemic and Epidemic Intelligence, Berlin, Germany; 2 World Health Organization, Geneva, Switzerland; 3 World Health Organization Regional Office for Africa, Brazzaville, Republic of Congo

**Keywords:** Public Health Intelligence, Influenza, Respiratory surveillance, Pandemic Preparedness, Epidemic Intelligence, Collaborative Surveillance, Viral Respiratory Events, Surveillance Systems Coordination, Cross-Sectoral Communication, Global Health Security

## Abstract

Recent public health emergencies, including the COVID-19 pandemic, MERS, and Avian Influenza outbreaks, underscore the need for effective surveillance systems for respiratory pathogens with epidemic and pandemic potential. In 2022, WHO initiated a project to help national public health professionals identify and address gaps in coordinating multiple surveillance systems for early detection and monitoring of viral respiratory events. The project involved developing country-specific approaches to address these gaps and identifying generalizable best practices. WHO headquarters collaborated with the WHO Regional Office for Africa (AFRO) to select three pilot countries: South Africa, Togo, and the United Republic of Tanzania. Each country conducted a landscape assessment of relevant surveillance activities followed by national workshops to discuss coordination, collaboration, and strengthening of Public Health Intelligence (PHI) for respiratory surveillance.

National workshops were held in Dar es Salaam (United Republic of Tanzania), Kpalimè (Togo), and Johannesburg (South Africa), bringing together professionals from various domains and sectors. The workshops highlighted system-specific and cross-cutting challenges and best practices related to respiratory surveillance. These findings informed a stakeholder workshop at the WHO Hub for Pandemic and Epidemic Intelligence in Berlin on 24–25 July 2024, which convened stakeholders from WHO headquarters, WHO AFRO, US CDC, and representatives from the pilot countries.

The workshop underscored the critical importance of coordination and collaboration in respiratory surveillance. By integrating multiple surveillance systems and fostering cross-sectoral communication, countries can enhance their ability to detect and respond to respiratory pathogens with epidemic and pandemic potential. The shared best practices and recommendations provide a valuable framework for strengthening global health security and preparedness.

## Background

Recent public health emergencies, including the COVID-19 pandemic, MERS, and Avian Influenza outbreaks in multiple countries around the globe highlight the need for effective surveillance systems for respiratory pathogens with epidemic and pandemic potential. As the World Health Organization (WHO) Mosaic Framework highlights, multiple fit-for-purpose surveillance systems are needed to address a country’s respiratory surveillance objectives [1]. In addition, capacities for collaboration are needed across systems within and beyond the health sector, to assess risks, generate robust insights, and inform decision-making. This is the concept of collaborative surveillance, which is designed to strengthen the global architecture for health emergency preparedness, response, and resilience (HEPR) [2, 3]. Different surveillance systems have their own strengths and weaknesses and may be optimized towards specific objectives related to early warning of emerging respiratory threats, monitoring and characterization of circulating pathogens, or assessment of the impact of health interventions. *Surveillance systems can work together collaboratively to achieve a common goal, while also functioning independently in a coordinated manner to contribute to that same goal.* A balance between collaboration and coordination is key to meeting the common goal efficiently. Improved coordination of different sources of surveillance information can improve the detection of and response to respiratory viruses with epidemic and pandemic potential, enabling more rapid risk assessment and improved decision-making. A coordinated approach is also crucial to pandemic preparedness, as it ensures the ability of teams to work together and more easily adapt surveillance platforms and products for future respiratory events.

In 2022, WHO launched a project to help national public health professionals identify and address gaps and challenges in coordinating multiple surveillance systems for the early detection and monitoring of viral respiratory events. The objectives were to develop country-specific approaches to address gaps in coordinating surveillance mechanisms and identify generalizable best practices. These efforts support and expand on other initiatives by aligning our work with the WHO’s Mosaic Framework and the HEPR global architecture, applying a Collaborative Surveillance approach, and leveraging the WHO Epidemic Intelligence from Open Sources (EIOS) initiative. WHO headquarters worked with the WHO Regional Office for Africa (AFRO) to select three pilot countries to engage in this work: South Africa, Togo, and the United Republic of Tanzania.

Each of the countries developed a landscape assessment of sentinel, event-based, and other relevant surveillance activities for respiratory pathogens and conducted a subsequent national workshop to discuss issues of coordination, collaboration, and overall strengthening of Public Health Intelligence (PHI) for respiratory surveillance. With support from WHO and the United States Centers for Disease Control and Prevention (CDC), national workshops on PHI for improving the detection of and response to influenza and other respiratory diseases were held in Dar es Salaam, United Republic of Tanzania, on 26–27 February 2024, in Kpalimè, Togo on 2–4 April 2024, and in Johannesburg, South Africa, on 7–8 May 2024. These workshops brought together professionals working on respiratory virus surveillance from different domains and sectors within each of the countries. The resulting workshop reports highlighted a combination of system-specific and cross-cutting challenges and best practices related to respiratory surveillance. The findings across the three countries were synthesized to identify common themes and used to inform the development of a stakeholder workshop at the WHO Hub for Pandemic and Epidemic Intelligence in July 2024 [8].

The WHO Hub for Pandemic and Epidemic Intelligence hosted a two-day workshop on 24–25 July 2024 in Berlin to convene stakeholders from WHO headquarters, WHO AFRO, US CDC, and representatives from the WHO country offices and national health authorities from South Africa, the United Republic of Tanzania, and Togo (Table 1). The aim of the meeting was to convene key stakeholders from each of the project pilot countries, foster collaboration, document challenges and best practices to coordinate across respiratory surveillance systems and identify opportunities and enablers to implement recommended practices. The workshop focused primarily on cross-system issues of coordination, communication, and collaboration while acknowledging the role played by system-specific challenges that were noted. This meeting report highlights the challenges to and considerations for coordinating respiratory surveillance systems identified during the workshop discussions.

**Table 1:** Technical contributors to the meeting

First Name	Last Name	Country / Organization
Adam	Crawley	US CDC
Aspen	Hammond	WHO headquarters (Global Influenza Programme)
Danstan	Ngenzi	Ministry of Health, United Republic of Tanzania
George Sie	Williams	WHO AFRO
Godwin	Akpan	WHO AFRO
Hannah	Lewis	WHO headquarters (Global Influenza Programme)
Jijoho Mischael Michel	Agbla	WHO AFRO
Joël Béni-Victorie	Anani	WHO Country Office, Togo
Kissaou	Kourkou Kpante	Ministry of Health, Togo
Lidia	Alexandrova	WHO headquarters (PHI)
Maria	Kelly	WHO Country Office, United Republic of Tanzania
Mbhekiseni	Khumalo	National Department of Health, South Africa
Mignon	du Plessis	National Institute of Communicable Diseases, South Africa
Mikfarou Tchamo	Kpegouni	Ministry of Health, Togo
Miriam	Matonya	Ministry of Health, United Republic of Tanzania
Ntsieni	Ramalwa-Sekhwama	WHO Country Office South Africa
Raquel	Medialdea Carrera	WHO headquarters (WHO Hub for Pandemic and Epidemic Intelligence)
Sharifa Sulelman	Mohamed	MoH Zanzibar, United Republic of Tanzania
Sibongile	Walaza	National Institute of Communicable Diseases, South Africa
Tomoka	Nakamura	WHO headquarters
Yeon Kyeng	Lee	WHO headquarters (WHO Hub for Pandemic and Epidemic Intelligence)
Zoulkarneiri	Issa	National Institute of Hygiene, Togo

## Sentinel and event-based surveillance systems generate complementary information but may be siloed

Event-based surveillance approaches, both at the community level and with open-source intelligence tools such as the EIOS system, are well positioned to provide early-warning for emerging respiratory threats [4]. Sentinel surveillance programs for influenza-like illness (ILI) and severe acute respiratory illness (SARI) syndromes are typically optimized to describe seasonality of respiratory viruses, signal activity at defined thresholds, and monitor virus types or lineages [5]. However, these surveillance activities are often siloed within health authorities which are housed in separate organizational units, supported by different staff, and use different methods and terminology. These challenges to coordination and collaboration persist across other surveillance activities and may hinder progress towards surveillance objectives. To address these broad challenges, workshop participants articulated key considerations in the areas of (1) coordination, (2) communication, and (3) data systems with a focus on respiratory surveillance.

## Coordination of respiratory surveillance activities

Efficient coordination and collaboration across systems and organizational units first requires awareness of the relevant stakeholders. Conducting a mapping exercise of surveillance systems that contribute to respiratory surveillance objectives, as well as relevant stakeholders involved in the implementation of those systems, can address this need. These may include sentinel, syndromic, notifiable disease, event-based, and other surveillance systems. System and stakeholder mapping may also support countries in applying the Mosaic Framework and strengthening further their collaborative surveillance approaches. However, awareness is not sufficient, and workshop participants articulated the need to identify and leverage platforms or mechanisms that can support coordination activities for respiratory surveillance. These might include routine in-person or virtual coordination meetings among points of contact for relevant surveillance systems, establishing email listservs among stakeholders, or creating WhatsApp groups for updates and alerting. Digital dashboards and web-based platforms may offer additional features that can enhance information-sharing. To guide and sustain these coordination activities, there may be a need to develop guidelines to formalize coordination across respiratory surveillance systems and stakeholders. Guidelines might include an up-to-date roster of key points-of-contact, establish the frequency of coordination meetings, establish aims or objectives, articulate procedures for data review and interpretation, and recommend indicators to monitor the impact of coordination and collaboration efforts. In addition to these efforts, the discussions also emphasized the critical need to further build and strengthen capacity for enhanced respiratory pathogen surveillance across Member States. This includes investing in training programs, developing standardized protocols, and ensuring the availability of necessary resources to support surveillance activities.

Once a country has mapped stakeholders and identified how they will coordinate and through which mechanisms, there is a need to identify and align relevant surveillance system outputs and information products, noting their frequency, key data points (e.g. % positivity; # signals verified), and responsible staff. Countries should evaluate which objectives (e.g. early warning; monitoring) these systems contribute to and which respiratory virus characteristics (e.g. transmissibility, severity, affected demographics) they describe and organize information products accordingly. Joint information products across systems and units may provide more context for decision-makers than individual surveillance system reports and may reduce duplicative efforts. Aligning these information products also creates an opportunity to develop standard operating procedures for the review and interpretation of multiple respiratory surveillance system outputs based on the country’s current capacities and systems.

## Communication of respiratory surveillance information and insights

Similar to internal mapping of surveillance systems, mapping the relevant stakeholders and audience(s) for receiving respiratory surveillance information products and alerts, including private sector and community actors, can strengthen collaboration and coordination. The information needs for each of these stakeholders should be articulated and categorized to ensure that only appropriate information is shared and in a relevant format. Roles and responsibilities for communication efforts should be articulated, including responsibilities for internal vs external communications and for development of risk communication and community engagement (RCCE) products. Language and messaging should reflect the intended audience, and communication focal points should engage directly with relevant subject matter experts (SMEs) to ensure that surveillance data are interpreted and communicated appropriately.

Mechanisms to receive and respond to stakeholder feedback, enabling two-way communication mechanisms with relevant internal and external stakeholders, may also yield benefits for public health agencies. Such an approach can support planning for and responding to “infodemics” related to respiratory threats using tools and approaches developed by WHO and other institutions [6].

## Surveillance and data systems

There may be opportunities to strengthen respiratory surveillance by leveraging or expanding existing systems and facilitating their integration rather than creating new systems, which may be more resource intensive. One example discussed by participants was the opportunity for countries to include ILI and/or SARI syndromes under notifiable or reporting disease surveillance structures, such as AFRO’s integrated disease surveillance and response (IDSR) framework, complementing sentinel surveillance systems with broader syndromic reporting for respiratory conditions. The use of alert thresholds can benefit the evaluation of information from surveillance systems, including both syndromic and sentinel systems, improving their usefulness for risk communication and decision-making [7]. WHO has recently updated the Pandemic Influenza Severity Assessment (PISA) guidance which provides a framework for systematically interpreting data collected through existing surveillance systems using thresholds and other contextual information.

Furthermore, countries may need to consider the establishment of electronic systems such as DHIS-2 which have the potential to support the visualization and interpretation of multiple data streams in context with one another, as well as automated alerting and reporting functionalities that reduce the burden on surveillance staff for routine tasks. As such platforms are developed, countries should explore what level of data integration is requested to support respiratory surveillance objectives. Notably, “integration” may be interpreted differently depending on the stakeholder and data can be (1) harmonized at the source of data collection for multiple uses; (2) linked across different surveillance systems; or (3) aggregated and/or compared during various analyses. In many cases, the third type of integration may be sufficient for reviewing different surveillance system outputs in context with one another.

Finally, to strengthen public health intelligence activities in Member States and enhance the use of the EIOS System, several key actions were outlined as recommendations. First, ensuring that EIOS system outputs continue to be included in the national and regional weekly Epi Bulletins and that relevant respiratory virus signals are triangulated and centralized along with other surveillance data. Additionally, establishing dedicated resources will allow EIOS system outputs to be fully leveraged and facilitate the coordination of insights drawn from multiple data sources. Integrating relevant signals detected in the EIOS system alongside other data and signals gathered through other data sources is also crucial. Furthermore, expanding the EIOS system to multiple ministries and agencies in the context of a One Health framework at the national level will enhance its reach and effectiveness. Finally, developing and implementing standard operating procedures (SOPs) for public health intelligence and EIOS system surveillance will ensure a standardized and efficient approach to surveillance activities.

In conclusion, the workshop underscored the critical importance of coordination and collaboration in respiratory surveillance. By integrating multiple surveillance systems and fostering cross-sectoral communication, countries can enhance their ability to detect and respond to respiratory pathogens with epidemic and pandemic potential. The shared best practices and recommendations from this workshop provide a valuable framework for strengthening global health security and preparedness (Table 1).

## Data Availability

Not applicable

## Abbreviations

AFRO: WHO Regional Office for Africa
EIOS: Epidemic Intelligence from Open Sources
HEPR: Health Emergency Preparedness, Response, and Resilience
IDSR: Integrated Disease Surveillance and Response
ILI: Influenza-Like Illness
MERS: Middle East Respiratory Syndrome
PHI: Public Health Intelligence
PISA: Pandemic Influenza Severity Assessment
RCCE: Risk Communication and Community Engagement
SARI: Severe Acute Respiratory Illness
SME: Subject Matter Expert
SOP: Standard Operating Procedure
WHO: World Health Organization
